# Caregivers of people with dementia and mental health during COVID-19: findings from a cross-sectional study

**DOI:** 10.1186/s12877-022-02752-x

**Published:** 2022-01-16

**Authors:** Anna Messina, Martina Lattanzi, Emiliano Albanese, Maddalena Fiordelli

**Affiliations:** Institute of Public Health, Faculty of Biomedical Sciences, Università della Svizzera Italiana, Lugano, Switzerland

**Keywords:** Caregivers, Dementia, Mental health, COVID-19, Cross-sectional study

## Abstract

**Background:**

There is sparse evidence on the impact on vulnerable populations of the COVID-19 pandemic. The aim of our study was to explore burden and mental wellbeing (including depressive, anxiety, and stress symptoms) in caregivers of people with dementia during the first wave of the pandemic in Italy and southern Switzerland, two bordering regions severely hit by the COVID-19 pandemic.

**Methods:**

We conducted an online cross-sectional survey with family carers of people with dementia between May and June 2020**.** We registered socio-demographic characteristics, and information about the relationship with the care recipient, dementia subtype, care inputs from others, and the need of care of the person with dementia. We measured caregiver burden with the Zarit Burden Interview (ZBI), psychological distress with the Depression, Anxiety and Stress Scale (DASS-21), and perceived isolation with the 3-item UCLA Loneliness Scale (UCLALS3).

**Results:**

Caregivers (*N* =571) reported moderate to severe care-related burden (mean=54.30; SD=18.33), moderate anxiety symptoms (mean=10.04; SD=6.93), mild depressive symptoms (mean=11.79; SD=6.12) and mild stress (mean=12.95; SD=5.53), and 72.3% of participants reported to feel lonely. All scores were significantly more severe in Swiss compared to Italian caregivers (all *p* values<0.001).

**Conclusions:**

We found that caregivers’ burden, anxiety symptoms, depression and perceived loneliness were marked during the first wave of the COVID-19 pandemic, in two severely hit bordering countries. Regional differences in the impact of the epidemic on caregivers could be due to contextual, societal, and cultural circumstances. As the pandemic endures, support to caregivers of people with dementia should be proportionate and tailored to needs and adapted to contextual factors.

## Introduction

Dementia influences those who are affected and the family members who very often care for their relatives at home [1]. Friends and relatives who provide non- professional and un-paid care to help a person, usually with long-term needs are defined as “informal carers” [2]. In 2019, according to the World Health Organization (WHO), informal caregivers worldwide spent over 89 billion hours assisting a family member with dementia in basic personal activities of daily living, with women contributing to 70% of global hours of care [3]. Informal care provision generally reflects more factors, from the scarcity or lack of resources and formal support services for people with dementia, to social and cultural expectations that family members, especially women, have the obligation to take care of a relative in need [4]. Even if there is evidence that caring duties can also lead to a sense of personal accomplishment and gratification [5], many informal caregivers deal with social, financial, and psychological strain that increases the likelihood of developing mental and physical distress [6].

Indeed, literature shows that caring for someone with dementia is associated with feelings of burden perceived stress, depression, loneliness, poorer immune function, and cognitive decline [7–10]. The COVID-19 pandemic brought new challenges to caregivers. Worldwide, governments enforced restrictions measures such as physical distancing, stay at home orders and travel restrictions that often limited the access to health-care facilities and caregivers support interventions such as respite services [3]. The disruption or abrupt suspension of social and medical support forced informal caregivers to take over multiple and additional responsibilities to meet the needs of the person with dementia [11]. In addition, since older age and premorbid conditions represent a risk factor for COVID 19 mortality [12], caregivers felt additional pressure to protect themselves from infection to prevent transmission to the person they cared for [13]. Taken together, all the changes imposed by the pandemic have likely exacerbated already taxing caring conditions and may have contributed to increase and worsen psychological distress of caregivers, potentially in the long term.

Longitudinal studies [14, 15] suggest that the impact on mental health on the general population varies through the waves and various phases of the COVID-19 pandemic, from outbreaks to relapses of mitigation public health measures, with marked geographic variations. However, there is scant evidence about caregivers’ mental health during the pandemic, particularly during the first wave of the outbreak and lockdown periods. Moreover, evidence on cross-country comparisons in psychological distress of caregivers is extremely thin [16], and inexistent from northern Italy and southern Switzerland, two regions that were very severely hit during the first pandemic outbreak. In addition, most of the few available studies on dementia caregivers during the pandemic, focused only on some psychological outcomes, mainly stress or caregiver burden [17–20] and varied in methods; psychological distress was often not measured using previously validated scales [21–23]. It is indispensable to expand and advance the current knowledge on the impact of the pandemic on caregivers of people with dementia to inform the design and provision of appropriate measures and interventions aimed at supporting this vulnerable population for the current and in view of future pandemic crises.

We conducted a cross-sectional survey in Italian and Swiss informal caregivers of people with dementia during the first wave of the COVID-19 pandemic (May- June 2020). We aimed to explore levels of burden, depressive symptoms, stress, anxiety, and perception of loneliness in caregivers of people with dementia during the first peak of the pandemic, in two hardly hit bordering countries, where preventive public health measures, including personal limitations and home confinement were strictly enforced. Public health preventive measures varied in the two countries, but according to the Oxford University’s stringency index were almost identical at the time of data collection of the present study [24].

## Methods

### Study participants and procedures

We conducted an online cross-sectional survey in family caregivers of people with dementia in two bordering, Italian-speaking regions: Italy and southern Switzerland**.** We used snowball technique to recruit a convenient sample of both Italian and Swiss informal caregivers by advertising our research via three different channels. An invitation message was prepared and circulated in 32 social media pages on Facebook and Instagram related to ageing and/or dementia and Facebook private groups of informal caregivers, and through 53 day-care centres for people with dementia in the two regions. Inclusion criteria were being 18 years of age or older, Italian-speaker, and informal (i.e. non professional) caregiver of a non-institutionalized family member with previously diagnosed dementia. Participants were excluded from the study if they did not match all inclusion criteria.

The online survey, implemented in RedCap (Research Electronic Data Capture), was active between May 25^th^, 2020 and June 25^th^, 2020. The estimated compilation time was 15 minutes. All participants received an informed consent to participate prior to filling out the survey, online. All methods were performed in accordance with the relevant guidelines and regulations.

## Measures

### Sociodemographic variables

We collected socio-demographic data of caregivers, including age, gender, place of residence, level of education, and work. We also asked carers about their relationship with the person with dementia, and inquired whether care provision was their only occupation, and if they received any care inputs from other formal or informal caregiver. Information about the care recipient elicited from the carers included the clinically diagnosed dementia subtype and level of autonomy in activities of daily living. All questions were asked and data collected in Italian.

### Psychological measures

All psychological measures were already available in Italian, were previously validated, and have been extensively used in Italian.

We used three main standardized questionnaires. The Italian version of the Zarit Burden Interview (ZBI) [25] to assess the level of caregiver burden. For each of the 22 items, respondents reported their perceived strain associated to the provision of care on a Likert scale ranging between zero (never) and four (nearly always). We computed total scores and applied standard cut-offs of low (<21), mild to moderate (21 ≤ x ≤ 40), moderate to severe (41 ≤ x ≤ 60), and severe burden (>60) [26]. The Italian short version of the Depression, Anxiety and Stress Scale (I-DASS-21) [27], to assess the mental health of caregivers. DASS-21 is commonly used to assess negative emotions in community samples, including in informal caregivers [28]. We changed and extended, from the original questionnaire delivery, the time reference of the items from “the past week” to “the past months of COVID-19 outbreak”. Respondents reported frequency of symptoms on a four-point Likert scale (never; sometimes; often; and almost always), and we calculated the separate scores of depressive, anxiety and stress-related symptoms (mild; moderate; severe; extremely severe) according to standard cut-offs [29]. Finally, we explored the frequency (hardly never; some of the time; often) of feelings of loneliness during the COVID-19 outbreak in the region, with the three items (lack of companionship, exclusion, and isolation) Italian version of the UCLA Loneliness scale (UCLALS3) [30, 31]. This scale has been previously used in population-based studies to measure social isolation, including in caregivers of people with dementia [32], and during the COVID-19 pandemic [33] . We asked participants to answer the items referring to the past months of COVID-19 outbreak. We computed an overall loneliness score, which ranged from three to nine, with higher scores indicating higher perception of loneliness.

### Statistical analyses

We computed means and proportions for descriptive statistics of the sociodemographic variables, Chi squared tests for all socio-demographic variables, and the main scales. We tested assumptions of normality and linearity, and we calculated correlations between the psychological distress measures, education, and years of caregiving experience using Pearson’s correlation coefficient and univariate and multivariate ANOVA regressions, setting statistical significance at 0.05. We assessed differences in ZBI, DASS-21, and UCLALS3 scores by country and sociodemographic characteristics using independent samples t-test. Finally, in a set of sensitivity analysis we ran linear regressions to model the effect of study site (i.e. Switzerland/ Italy) separately on each of the psychological distress scores adjusting for relevant socio-demographic and care characteristics. We used SPSS 25.0 statistical software for Windows for all statistical analyses.

## Results

### Sociodemographic characteristics

Of the 646 caregivers contacted, 571 completed the survey and formed the analytic sample (response rate 87%). Table 1 shows the sociodemographic characteristics of the overall study sample, and by country. Of the 571 caregivers, 425 were Italian (74.4%) and 146 (25.6%) were Swiss, with a mean age of 53 years (SD=11.99) and a range of 24 to 89. The majority of caregivers were female (81.6%), and the mean number of years spent in caregiving was 6 (SD=3.95). Most participants cared for a family member affected by Alzheimer’s Disease (55.3%), followed by Vascular (16.6%), Parkinson’s (12.6%), Frontotemporal dementia (7.7%), Lewy-Body dementia (3.3%), and other or unspecified types of dementia (4.5%). Most caregivers were children (71.8%), or spouse of the person with dementia (20.7%) and referred to care for a person not autonomous in most daily life activities (79.9%). Almost two thirds of participants admitted getting help from others in caring (58.7%), especially from other family members (32.2%) or professional carers (22.8%). More than half of participants had at least higher secondary education (56.4%), and almost half of the caregivers were employed with a full time or part-time job (49.6%).

**Table 1:** Sociodemographic characteristics of informal caregivers by country (*N* = 571)

	Total sample (*N* = 571)	Italy (*N* = 425)	Switzerland, Ticino (*N* = 146)	
Variable	N (%)	N (%)	N (%)	***p***-value*
**Gender**				
Female	466 (81.6)	381 (89.6)	85 (58.2)	*p*<0.001
Male	104 (18.2)	43 (10.1)	61 (41.8)	
Not specified	1 (0.2)	1 (0.2)		
				*p*-value**
**Age Mean (SD)**	53.54 (11.99)	51.85 (10.72)	58.49 (13.99)	*p*<0.001
**Years of caregiving Mean (SD)**	6.07 (3.95)	5.08 (3.68)	8.83 (3.31)	*p*<0.001
**Caregiver as sole occupation**				
Yes	292 (51.1)	176 (41.4)	116 (79.5)	*p*<0.001
No	279 (51.1)	249 (58.6)	30 (20.5)	
**Employment Status**				
Unemployed/Housewife	202 (35.4)	132 (31.1)	70 (47.9)	*p*<0.001
Full-time Job	253 (44.3)	228 (53.6)	25 (17.1)	
Part-time Job	30 (5.3)	25 (5.9)	5 (3.4)	
Retired	86 (15.1)	40 (9.4)	46 (31.5)	
**Relationship with the care-recipient**				
Child	410 (71.8)	328 (77.2)	82 (56.2)	*p*<0.001
Spouse	118 (20.7)	59 (13.9)	59 (40.4)	
Other	43 (7.5)	38 (8.9)	5 (3.4)	
**Education**				
Compulsory education	107 (18.7)	70 (16.5)	37 (25.3)	*p*<0.001
Higher Secondary education	322 (56.4)	231 (54.4)	91 (62.3)	
University education	142 (24.9)	124 (29.2)	18 (12.3)	
**Care recipient type of dementia**				
Alzheimer	316 (55.3)	247 (58.1)	69 (47.3)	*p*<0.001
Vascular dementia	95 (16.6)	82 (19.3)	13 (8.9)	
Parkinson's Disease	72 (12.6)	24 (5.6)	48 (32.9)	
Frontotemporal dementia	44 (7.7)	44 (10.4)	0	
Dementia with Lewy Bodies	19 (3.3)	17 (4.0)	2 (1.4)	
Other	25 (4.5)	11 (2.6)	14 (9.6)	
**Autonomy in basic function of the care recipient**				
Yes	116 (20.3)	105 (24.7)	11 (7.5)	*p*<0.001
No	455 (79.7)	320 (75.3)	135 (92.5)	
**Help from others**				
Yes	335 (58.7)	285 (67.1)	50 (34.2)	*p* = 0.197
Relative	184 (32.2)	159 (55.8)	25 (50.0)	
Professional carers (nurse/domestic worker)	130 (22.8)	106 (37.2)	24 (48.0)	
Friends/neighbours	21 (3.7)	20 (7.0)	1 (2.0)	
No	236 (41.3)	140 (32.9)	96 (65.8)	

*P* values were calculating using *Chi Squared tests, and **Independent t-test, as appropriate

### Psychological measures

**Fig. 1:**
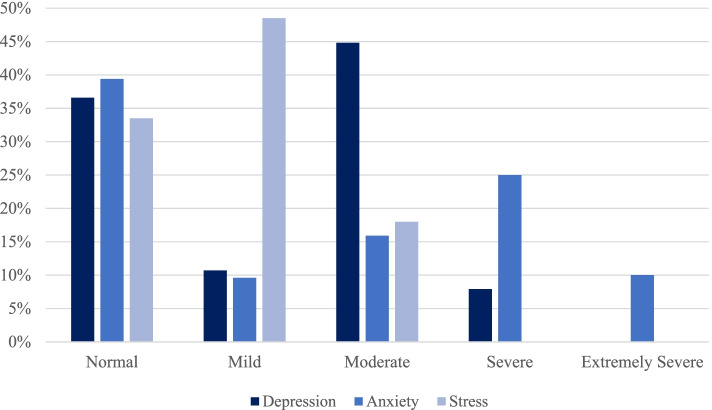
Proportion (%) of participants with depressive, anxiety, and stress (DASS-21) symptoms according to severity. Cut-offs scores [29]: Depression (normal: 0-9, mild: 10-12, moderate: 13-20, severe: 21-27, extremely severe: 28-42); Anxiety (normal: 0-6, mild: 7-9, moderate: 10-14, severe 15-19, extremely severe: 20-42); Stress (normal: 0-10, mild: 11-18, moderate: 19-26, severe: 27-34, extremely severe: 35-42)

Overall, caregivers had a mean Zarit burden score of 54.3 (SD=18.3), which corresponds to “moderate to severe”. Mean scores from DASS-21 showed mild depression (mean=11.79; SD=6.12), moderate anxiety (mean=10.04; SD=6.93), and mild stress (mean=12.95; SD=5.53), according to standard cut-offs [29]. However, the severity of reported symptoms was more pronounced for anxiety, intermediate for depression, and less marked for stress (Fig. 1). According to past categorizations of the UCLALS3 scale [34], 72.3% of caregivers fell into the ‘lonely’ category, 99.3% among Swiss and 63.1% in Italian caregivers.

All psychological variables were positively correlated to each other (r^2^ values ranging from 0.59 to 0.85, all *p* values <0.001) (Table 2). Correlations did not vary between countries and are presented for the full sample. More specifically, multiple linear regressions showed that stress were significantly associated with depression levels (β=0.52; *p*<0.001) and burden (β=0.43; *p*<0.001); depression explained a significant proportion of variance in perception of loneliness (β=0.49; *p*<0.001), anxiety (β=0.42; *p*<0.001), and stress scores (β=0.53; *p*<0.001); anxiety was significantly associated with depression (β=0.33; *p*<0.001), loneliness (β=0.27; *p*<0.001) and stress (β=0.26; *p*<0.001); perception of loneliness was significantly associated with symptoms of depression (β=0.17; *p*<0.001) and anxiety (β=0.12; *p*<0.001). In addition, higher levels of education and years of experience in caregiving slightly predicted better mental health on all outcomes (all *p* values <0.001) (Table 2).

**Table 2:** Multiple linear regressions for psychological variables

Variable	R	*R*^2^ (*p*-value)	Caregiver burden	Depression	Anxiety	Stress	Loneliness	Education	Years of experience
Caregiver burden	0.79	0.62 (<0.001)		0.08 (0.20)	0.20 (0.16)	0.43 (0.20)***	0.02 (0.36)	-0.09 (0.31)**	0.11 (0.13)***
Depression	0.92	0.85 (<0.001)	0.03 (0.01)		0.33 (0.03) ***	0.52 (0.04)***	0.17 (0.07)***	-0.00 (0.06)	-0.25 (0.03)
Anxiety	0.90	0.82 (<0.001)	0.10 (0.01)**	0.42 (0.05)***		0.30 (0.05)*	0.12 (0.09)***	-0.07 (0.08)**	0.05 (0.03)*
Stress	0.91	0.84 (<0.001)	0.19 (0.01)*	0.53 (0.03)***	0.26 (0.03)***		0.00 (0.07)	0.06 (0.06)**	0.02 (0.03)
Loneliness	0.77	0.59 (<0.001)	0.03(0.00)***	0.49 (0.02)***	0.27 (0.02)***	0.01 (0.02)		-0.02 (0.04)	0.01 (0.02)

Beta standardized coefficients (*β*) are reported with Beta standard errors in parentheses

*, **, *** indicates *p* values <0.05, <0.005, <0.001, respectively

Independent t-test showed that all psychological symptoms were more pervasive in Swiss caregivers with higher scores for burden depression, stress, anxiety, and loneliness than the Italian counterparts (all *p* values < 0.001) (Table 3).

**Table 3:** Comparison of psychological variables between Swiss (*N* =146) and Italian (*N* =425) caregivers

Variable	All sample Mean (SD) [min/max]	Italians Mean (SD) [min/max]	Swiss Mean (SD) [min/max]	T statistics* (df)	***P***-value
**ZBI**					
Caregiver burden	54.30 (18.33) [6-88]	48.75 (16.90) [6-88]	70.46 (11.48) [33-88]	-14.41 (569)	<0.001
**DASS-21**					
Depression	11.79 (6.12) [0-21]	10.23 (6.17) [0-21]	16.36 (2.76) [10–21]	-11.61 (569)	<0.001
Anxiety	10.04 (6.93) [0-21]	7.82 (6.48) [0-21]	16.50 (3.05) [4–21]	-15.59 (569)	<0.001
Stress	12.95 (5.53) [0-21]	11.60 (5.60) [0-21]	16.89 (2.71) [8–21]	-10.97 (569)	<0.001
**UCLALS3**					
Loneliness	6.77 (2.06) [3–9]	6.29 (0.10) [3–9]	8.20 (0.79) [5–9]	-10.62 (569)	<0.001

*ZBI* Zarit Burden Interview (possible range, 6 to 88), *DASS-21* Depression Anxiety and Stress Scale (possible range, 0-21), *UCLALS3* Ucla Loneliness Scale 3 items version (possible range, 3-9)

* Students’ t test for independent samples

*P* values were calculated using *Chi Squared tests, and *Independent t-test, as appropriate

The linear regression models of the effect of country of residence on the psychological distress measures confirmed that anxiety, depression, stress, and burden were, respectively, 55% (β=0.547), 44% (β=0.438), 42% (β=0.418), and 52% (β=0.517) higher in Switzerland compared to Italy (all *p* values < 0.001). All associations remained significant also after we adjusted for relevant covariates (all *p* values < 0.001).

We also found significant differences for social support, with caregivers who got help from others showing lower levels of burden (mean=49.27; SD=16.41), depression (mean=10.66; SD=6.02), loneliness (mean=6.34; SD=2.08) and anxiety (mean=8.65; SD=6.65) than caregivers who took care of their relatives alone (mean=13.40; SD=5.89); (mean=7.39; SD=1.85); (mean=12.02; SD=6.84) (all *p* values < 0.001). Similarly, spouses’ caregivers reported significantly higher scores in all psychological outcomes than children caregivers (all *p* values < 0.001).

## Discussion

Our study aimed to explore psychological distress in carers of people with dementia during the first wave of COVID-19 pandemic in two severely hit bordering countries. We found that levels of burden, anxiety, depression, and perception of loneliness were marked in caregivers of people with dementia. All psychological symptoms, including loneliness were positively correlated to each other, and were more pervasive in Swiss compared to Italian caregivers, and in spouse compared to children caregivers. Education, employment status and social support were inversely associated with psychological distress.

Previous evidence suggested that providing care for a person with dementia can cause strain and can affect both the psychological and physical health of the carer [8]. Caregivers generally report higher levels of perceived stress, depression, loneliness, burden and lower levels of self-efficacy and well-being compared to the general population [7]. Our results on the inverse association between educational level, social support, employment status, and psychological distress are consistent with those of pre-pandemic studies [35]. Nevertheless, disruption of healthcare facilities and social restriction measures imposed by the pandemic altered care routines and practices, with plausible detrimental consequences on caregivers’ physical and mental health [11]. Direct comparisons with literature are not straightforward because few studies have been conducted on caregivers of people with dementia since the beginning of the pandemic, and in particular during the lockdown, in spring 2020 [36]. However, previous studies [37–39] found that high variations in social support services were associated with increased levels of anxiety, burden, and depression in samples of caregivers from Italy and the UK respectively.

We found some variations in the reported psychological symptoms. While severe levels of reported burden and anxiety were common, more than half of the caregivers in our sample reported only mild to moderate levels of stress and depressive symptoms. The COVID-19 pandemic may affect caregivers’ mental health on multiple levels. The disruption of health care services may led to a sudden and unexpected increase in responsibility. Caregivers had to provide more types of care inputs and for longer hours, with a consequent lower sense of competence and mastery [40], which in turn are associated to a greater experience of burden [41]. Suspension of respite care and breaks may have altered coping mechanisms and pre-pandemic recover opportunities, contributing to “chronic stressor felt by caregiver with respect to physical and emotional well-being, family relations and financial status” [42], rather than the more transitory experience of stress to which caregivers can adapt over time. In addition, fear of infecting a loved one can trigger worry and anxiety [43]. Moreover, the uncertainties about infection risks and the very limited knowledge about COVID-19, especially during the first wave of the pandemic, were likely responsible of increases in cognitive alertness, which may shadow or counterbalance depressive symptoms or their perception. Indeed, depression rates in the general population increased during the second wave of the pandemic compared to the first wave, probably because of the prolonged psychological distress and long-term social dislocations [44].

In our study, we also focused on perceived loneliness. We found that most caregivers reported to feel lonely. This may be explained by the almost complete lack of social interactions imposed by restriction measures, which was abrupt and unprecedented. The reported feeling of being alone and trapped at home with few external support was probably consequent to an actual condition of forced confinement [45]. These findings suggest that caregivers of people with dementia are likely vulnerable to lockdown and social restriction measures, and could suffer remarkable loneliness, which may compromise their ability to provide care [46]. Next, psychological measures correlated to each other, correlations between depression and anxiety were particularly strong, and with higher scores in reported anxiety, predicting higher scores in depression and vice versa. Since a third of our sample reported from severe to extremely severe levels of anxiety, there is a concrete risk of increasing levels of depression in caregivers as the pandemic endures.

We investigated mental health in caregivers during the pandemic in two different countries and Swiss caregivers reported significantly higher distress in all mental health outcomes than their Italian counterparts did. During the first wave, the Italian Government adopted slightly stricter public health measures to contain the pandemic compared to the Swiss Confederation [24]. The extent to which and potential causal role of preventive measures on caregivers’ mental health are not easy to disentangle. However, the variation in the timing and severity of the restriction measures adopted in the two countries may have contributed, at least to some extent, to explain the differences we found between Swiss and Italian caregivers. The presence of prompt preventive measures to reduce the risk of infection, especially for the elderly and vulnerable populations, may have contributed to lessen the anxiety and fear of contagion in Italian caregivers of people with dementia. On the other hand, the latency and minor severity of restrictions adopted by the Swiss Federal Council may have triggered a sense of personal unsafety for both the caregiver and the care-recipient. Nonetheless, other socio-cultural and contextual factors may contribute to explain the fact that Swiss caregivers reported significantly higher distress in all mental health outcomes than Italian carers did. Evidence suggests that social support is a protective factor towards burden and psychological distress in dementia caregivers [47, 48]. Since in our study Italian caregivers reported to receive more help in caring duties compared to Swiss caregivers (Table 1), the differences in use and availability of emotional and practical forms of support during the pandemic may lead the burden due to the disruption of services and the additional care responsibilities. A further investigation on specific restrictive measures and services available during the lockdown for caregivers of people with dementia in the two different countries is needed to clarify the differences.

The present study is not free from limitations. The lack of longitudinal or pre-pandemic data on the burden, mental health, and loneliness of caregivers limits causal inference. However, we found exceptionally high levels of burden and psychological distress according to commonly used and standardized scales. Further, although the sample of our study was large it was not representative of the target population. We cannot exclude selection bias also because only caregivers who had access to the internet and to our recruitment channels could participate in the survey. Nevertheless, the study population had a broad sociodemographic spectrum, which provides support at least to some extent to the external validity of our results. We measured psychological distress using robust and valid measures, and participants self-reported a wide range of their socio-demographic and care characteristics. However, people with dementia were less thoroughly characterised. We did not measure behavioural and psychological symptoms of dementia (BPSD), and we used a binary question to measure autonomy and not a standard measure of activities of daily living (ADL). BPSD and ADL impairments are associated with strain and psychological distress in caregivers [49, 50], and may have worsened because of self-sheltering, quarantine, and other personal and social restrictive measures [51].

Our results confirmed that people with dementia and their caregivers have faced serious challenges during the pandemic. Local authorities must consider, locally adapt, and apply the recommendations of issued by the Tecnhical Advisory Board on Mental Health in the WHO European region to reduce the impact of COVID-19 crisis on mental health in vulnerable populations [52].

## Conclusions

The present study showed that family caregivers of people with dementia have experienced psychological distress during the first wave of COVID-19 pandemic. Since we found severe feelings of burden and anxiety, rapid and targeted measures are required to enable carers to continue provide care and cope with uncertainty, while maintaining their own well-being. Further interventions should address feelings of loneliness accounting for contextual and cultural circumstances.

## Data Availability

The dataset generated and analysed during the current study are available in the Zenodo open access repository, DOI:10.5281/zenodo.4748652.

## References

[1] Alzheimer’s Disease International (ADI) (2018). Global estimates of informal care.

[2] Health at a Glance 2017. OECD; 2017.

[3] Health Organization W (2021). Global status report on the public health response to dementia [Internet].

[4] Mccleary L, Blain J (2013). Cultural values and family caregiving for persons with dementia. Indian J Gerontol.

[5] Yu DSF, Cheng S-T, Wang J (2018). Unravelling positive aspects of caregiving in dementia: an integrative review of research literature. Int J Nurs Stud.

[6] Bullock R. The needs of the caregiver in the long-term treatment of Alzheimer disease. Alzheimer Dis Assoc Disord. 2004;18:17–23. 10.1097/01.wad.0000127493.65032.9a 15249844

[7] Schulz R, Martire LM (2004). Family caregiving of persons with dementia: prevalence, health effects, and support strategies. Am J Geriatr Psychiatry.

[8] Gilhooly KJ, Gilhooly MLM, Sullivan MP, McIntyre A, Wilson L, Harding E, et al. A meta-review of stress, coping and interventions in dementia and dementia caregiving. BMC Geriatr. 2016;16:106. 10.1186/s12877-016-0280-8 PMC4872341 27193287

[9] Kovaleva M, Spangler S, Clevenger C, Hepburn K. ChronicStress_SocialIsolation_Caregivers. 2017;1–8.

[10] Vitaliano PP, Zhang J, Scanlan JM (2003). Is caregiving hazardous to one’s physical health? A meta-analysis. Psychol Bull.

[11] Wang H, Li T, Barbarino P, Gauthier S, Brodaty H, Molinuevo JL (2020). Dementia care during COVID-19. Lancet.

[12] O’Shea E. Remembering people with dementia during the COVID-19 crisis [version 2; peer review: 4 approved]. HRB Open Res. 2020;3:15. Available from: https://hrbopenresearch.org/articles/3-15/v2. 10.12688/hrbopenres.13030.1 PMC7195897 32510035

[13] Bonanad C, García-Blas S, Tarazona-Santabalbina FJ, Díez-Villanueva P, Ayesta A, Sanchis Forés J (2020). Coronavirus: the geriatric emergency of 2020. Joint document of the Section on Geriatric Cardiology of the Spanish Society of Cardiology and the Spanish Society of Geriatrics and Gerontology. Rev Esp Cardiol.

[14] Ramiz L, Contrand B, Rojas Castro MY, Dupuy M, Lu L, Sztal-Kutas C (2021). A longitudinal study of mental health before and during COVID-19 lockdown in the French population. Glob Health.

[15] Pierce M, McManus S, Hope H, Hotopf M, Ford T, Hatch SL, et al. Mental health responses to the COVID-19 pandemic: a latent class trajectory analysis using longitudinal UK data. Lancet Psychiatry. 2021. 10.1016/S2215-0366(21)00151-6. 10.1016/S2215-0366(21)00151-6 PMC9764381 33965057

[16] Bergmann M, Wagner M (2021). The impact of COVID-19 on informal caregiving and care receiving across Europe during the first phase of the pandemic. Front Public Health.

[17] Giebel C, Cannon J, Hanna K, Butchard S, Eley R, Gaughan A, et al. Impact of COVID-19 related social support service closures on people with dementia and unpaid carers: a qualitative study. Aging Ment Health. 2020;25(7):1281–8. 10.1080/13607863.2020.1822292 32954794

[18] Cohen G, Russo MJ, Campos JA, Allegri RF. Living with dementia: increased level of caregiver stress in times of COVID-19. Int Psychogeriatr. 2020;32(11):1377–81. 10.1017/S1041610220001593 PMC7453351 32729446

[19] Park SS (2021). Caregivers’ mental health and somatic symptoms during COVID-19. J Gerontol Ser B Psychol Sci Soc Sci.

[20] Borelli WV, Augustin MC, de Oliveira PBF, Reggiani LC, Bandeira-de-Mello RG, Schumacher-Schuh AF (2021). Neuropsychiatric symptoms in patients with dementia associated with increased psychological distress in caregivers during the COVID-19 pandemic. J Alzheimers Dis.

[21] Carcavilla N, Pozo AS, Gonzalez B, Moral-Cuesta D, Roldan JJ, Erice V (2021). Needs of dementia family caregivers in Spain during the COVID-19 pandemic. J Alzheimers Dis.

[22] Canevelli M, Valletta M, Toccaceli Blasi M, Remoli G, Sarti G, Nuti F (2020). Facing dementia during the COVID-19 Outbreak. J Am Geriatr Soc.

[23] Tsapanou A, Papatriantafyllou JD, Yiannopoulou K, Sali D, Kalligerou F, Ntanasi E (2021). The impact of COVID-19 pandemic on people with mild cognitive impairment/dementia and on their caregivers. Int J Geriatr Psychiatry.

[24] Hale T, Angrist N, Goldszmidt R, Kira B, Petherick A, Phillips T (2021). A global panel database of pandemic policies (Oxford COVID-19 government response tracker). Nat Hum Behav.

[25] Chattat R, Cortesi V, Izzicupo F, del Re ML, Sgarbi C, Fabbo A (2011). The Italian version of the Zarit Burden interview: a validation study. Int Psychogeriatr.

[26] Zarit SH, Reever KE, Bach-Peterson J (1980). Relatives of the impaired elderly: correlates of feelings of burden. The Gerontologist.

[27] Bottesi G, Ghisi M, Altoè G, Conforti E, Melli G, Sica C (2015). The Italian version of the depression anxiety stress scales-21: factor structure and psychometric properties on community and clinical samples. Compr Psychiatry.

[28] Ahmad Zubaidi ZS, Ariffin F, Oun CTC, Katiman D (2020). Caregiver burden among informal caregivers in the largest specialized palliative care unit in Malaysia: a cross sectional study. BMC Palliat Care.

[29] Lovibond SH, Lovibond PF. Manual for the depression anxiety stress scales. (2nd ed.). ed; 1995.

[30] Hughes ME, Waite LJ, Hawkley LC, Cacioppo JT (2004). A short scale for measuring loneliness in large surveys: results from two population-based studies. Res Aging.

[31] Solano L, Coda R (1994). Relazioni, emozioni, salute. Introduzione alla psicoimmunologia. [Relationships, emotions and health: Introduction to psychoimmunology] [Internet].

[32] Rand S, Malley J, Vadean F, Forder J (2019). Measuring the outcomes of long-term care for unpaid carers: comparing the ASCOT-carer, carer experience scale and EQ-5D-3 L. Health Qual Life Outcomes.

[33] Groarke JM, Berry E, Graham-Wisener L, McKenna-Plumley PE, McGlinchey E, Armour C (2020). Loneliness in the UK during the COVID-19 pandemic: cross-sectional results from the COVID-19 Psychological Wellbeing Study. PLoS ONE.

[34] Steptoe A, Shankar A, Demakakos P, Wardle J (2013). Social isolation, loneliness, and all-cause mortality in older men and women. Proc Natl Acad Sci.

[35] Pinquart M, Sörensen S (2003). Differences between caregivers and noncaregivers in psychological health and physical health: a meta-analysis. Psychol Aging.

[36] Greenberg NE, Wallick A, Brown LM. Impact of COVID-19 pandemic restrictions on community-dwelling caregivers and persons with dementia. Psychol Trauma Theory Res Pract Policy. 2020;12(S1):220–1. 10.1037/tra0000793 32584105

[37] Giebel C, Lord K, Cooper C, Shenton J, Cannon J, Pulford D, et al. A UK survey of COVID-19 related social support closures and their effects on older people, people with dementia, and carers. Int J Geriatr Psychiatry. 2020;36(3):393–402. 10.1002/gps.5434 PMC7536967 32946619

[38] Altieri M, Santangelo G. The psychological impact of COVID-19 pandemic and lockdown on caregivers of people with dementia. Am J Geriatr Psychiatry. 2021;29(1):27–34. 10.1016/j.jagp.2020.10.009 PMC7577876 33153872

[39] Carpinelli Mazzi M, Iavarone A, Musella C, de Luca M, de Vita D, Branciforte S, et al. Time of isolation, education and gender influence the psychological outcome during COVID-19 lockdown in caregivers of patients with dementia. Eur Geriatr Med. 2020;11(6):1095–8. 10.1007/s41999-020-00413-z PMC7556578 33052535

[40] Phillips D, Paul G, Fahy M, Dowling-Hetherington L, Kroll T, Moloney B (2020). The invisible workforce during the COVID-19 pandemic: family carers at the frontline. HRB Open Res.

[41] Chan E-Y, Glass G, Chua K-C, Ali N, Lim W-S (2018). Relationship between mastery and caregiving competence in protecting against burden, anxiety and depression among caregivers of frail older adults. J Nutr Health Aging.

[42] Pearlin LI, Mullan JT, Semple SJ, Skaff MM (1990). Caregiving and the stress process: an overview of concepts and their measures 1 [Internet].

[43] Amsalem D, Dixon LB, Neria Y (2021). The Coronavirus disease 2019 (COVID-19) outbreak and mental health: current risks and recommended actions. JAMA Psychiatry.

[44] Fukase Y, Ichikura K, Murase H, Tagaya H (2021). Depression, risk factors, and coping strategies in the context of social dislocations resulting from the second wave of COVID-19 in Japan. BMC Psychiatry.

[45] van Wijngaarden E, van der Wedden H, Henning Z, Komen R, The A-M (2018). Entangled in uncertainty: The experience of living with dementia from the perspective of family caregivers. PLoS ONE.

[46] Quinn C, Nelis SM, Martyr A, Morris RG, Victor C, Clare L (2020). Caregiver influences on ‘living well’ for people with dementia: Findings from the IDEAL study. Aging Ment Health.

[47] Adelman RD, Tmanova LL, Delgado D, Dion S, Lachs MS. Caregiver burden: a clinical review. JAMA. 2014;311(10):1052–60. 10.1001/jama.2014.304 24618967

[48] Rodakowski J, Skidmore ER, Rogers JC, Schulz R. Role of social support in predicting caregiver burden. Arch Phys Med Rehabil. 2012;93(12):2229-36. 10.1016/j.apmr.2012.07.004 PMC3508254 22824248

[49] Kim B, Noh GO, Kim K (2021). Behavioural and psychological symptoms of dementia in patients with Alzheimer’s disease and family caregiver burden: a path analysis. BMC Geriatr.

[50] Kang HS, Myung W, Na DL, Kim SY, Lee J-H, Han S-H (2014). Factors associated with caregiver burden in patients with Alzheimer’s disease. Psychiatry Investig.

[51] Pongan E, Dorey J-M, Borg C, Getenet JC, Bachelet R, Lourioux C (2021). COVID-19: association between increase of behavioral and psychological symptoms of dementia during lockdown and caregivers’ poor mental health. J Alzheimers Dis.

[52] Health Organization W (2021). Action required to address the impacts of the COVID-19 pandemic on mental health and service delivery systems in the WHO European Region: recommendations from the European Technical Advisory Group on the Mental Health Impacts of COVID-19 [Internet].

